# Hemispheric asymmetries in word recognition as revealed by the orthographic uniqueness point effect

**DOI:** 10.3389/fpsyg.2014.00244

**Published:** 2014-03-21

**Authors:** Cristina Izura, Victoria C. Wright, Nathalie Fouquet

**Affiliations:** ^1^Department of Psychology, Swansea UniversitySwansea, UK; ^2^Department of Psychology, Aberystwyth UniversityAberystwyth, UK

**Keywords:** orthographic uniqueness point, visual word recognition, cerebral hemispheres, N170, serial/parallel processing, event-related potential

## Abstract

The orthographic uniqueness point (OUP) refers to the first letter of a word that, reading from left to right, makes the word unique. It has recently been proposed that OUPs might be relevant in word recognition and their influence could inform the long-lasting debate of whether – and to what extent – printed words are recognized serially or in parallel. The present study represents the first investigation of the neural and behavioral effects of OUP on visual word recognition. Behaviourally, late OUP words were identified faster and more accurately in a lexical decision task. Analysis of event-related potentials demonstrated a hemispheric asymmetry on the N170 component, with the left hemisphere appearing to be more sensitive to the position of the OUP within a word than the right hemisphere. These results suggest that processing of centrally presented words is likely to occur in a partially parallel manner, as an ends-in scanning process.

## INTRODUCTION

The orthographic uniqueness point (OUP) of a printed word is the letter position, starting from the left, at which the word is distinguishable from all other words in the mental lexicon. For example, the OUP of “*acrylic”* is four. This reflects the fact that, when reading the word “*acrylic”* from left to right, upon reading the letter “*y,”* “*acrylic”* is the only possible remaining match. By the same token, the OUP of “*brother”* is 7 as, at letter position 6, there are still other possible matches such as for example “*brothel*.” The OUP of words has been proposed as a major determinant of the moment in time in which words are recognized (Kwantes and Mewhort, 1999). If this proves to be the case, our understanding of how printed material is processed will move forward in an unexpected direction. The evidence to date is unclear since the few studies exploring the effect of OUP on the recognition of single words have shown mixed results.

Kwantes and Mewhort (1999) were the first to study the potential influence of OUP in word naming. They found that, on average, words with early OUPs were named 26 ms faster than words with late OUPs, concluding that visual word recognition proceeds in a highly sequential manner. A few years later Lindell et al. (2003) investigated whether this sequential processing of words could be applied to both hemispheres, since according to some accounts, such as the dual mode hypothesis, only serial mechanisms of word processing are available to the right hemisphere while the left hemisphere is endowed with an extra and efficient parallel processing system (Ellis and Young, 1985; Bub and Lewine, 1988; Ellis et al., 2009). Lindell et al. (2003) presented the same 7-letter early and late OUP words used by Kwantes and Mewhort (1999), to the left and right visual fields (RVFs) within the context of a lexical decision task. They found a 33 ms advantage for early over late OUP words with no interactions leading them to conclude that both hemispheres process words in a serial manner. These findings were replicated in a follow-up study by the same group (Lindell et al., 2005), where they assessed the performance of each of the hemispheres when naming laterally presented early and late OUP words. Early OUP were named faster than late OUP in the LH but not in the RH (Experiment 1), the lack of OUP effect in the RH was attributed to the relatively poor perceptibility of the initial letters of words presented in the left visual field (LVF).

The role that the beginning of words plays on word processing has also been studied in relation to the parafoveal information available during fluent reading. The measure used here has not been the OUP but the degree to which the first three letters of the word constraint the number of potential target words. High-constraint words first letters generate few words (e.g., *tyrant, awkward*) while low-constraint words start with letters shared with many other words (e.g., *climax, scrawny*). Hand et al. (2012) found facilitated processing for parafoveal previewed targets with high constraining initial letters. This is assumed to be related to the fact that, during reading, the perceptual span is such that processing of words is not restricted to the currently fixated word and that processing of a parafoveal word begins before fixation (McConkie and Rayner, 1975). Thus, the processing of high-constraint words was facilitated since they generate fewer target candidates than low-constraint words. Rayner et al. (1982) also demonstrated that an invalid parafoveal preview impaired performance when compared with a valid preview, highlighting the importance of the initial letters in reading. The effect of OUP has also been investigated in relation to the parafoveal preview benefit. Miller et al. (2006) used a sentence boundary reading task with eye-tracking measures. Target words were matched for a range of variables, including frequency of the initial trigram. Three preview conditions were included: no parafoveal preview (e.g., *baby thqjzwp*), partial preview (e.g., *baby girazwp*), and full preview (e.g., *baby giraffe*). It was argued that if words are read in a serial-like manner an advantage for early OUP targets would be observed, as the extent of the preview (three letters) corresponded with the position of early OUP words. Strikingly, Miller et al. (2006) found no benefit for early OUP words but a small and reliable advantage for late OUP words. This is consistent with findings of a faster processing of low-constraint words (Lima and Inhoff, 1985) and opposite to the pattern of results reported by Kwantes and Mewhort (1999), Lindell et al. (2003, 2005).

Lamberts (2005) argued that a potential account for these mixed results is that the observed OUP effects were confounded with total lexical overlap, a factor controlled in Miller et al.’s (2006) study. Total lexical overlap refers to the number of letters-in-position shared by the target and other words within the lexicon. For example, *house* and *goose* share three letters-in-position in common. In a computational analysis, Lamberts (2005) found that Kwantes and Mewhort’s (1999) early OUP stimuli shared four letters-in-position with 19 other words in the database; by contrast, late OUP words shared four letters-in-position with 46 other words. Thus, the OUP effects reported by Kwantes and Mewhort (1999) may have been confounded with the extent to which words with early and late OUPs overlapped with other lexical entries rather than the impact of the position of the uniqueness point. More recently, another measure of lexical overlap has been proposed as a better way of operationalising orthographic similarity. This is the orthographic Levenshtein distance 20 (OLD20) which is a measure of the minimum number of additions, subtractions and substitutions required to produce a word from another (Yarkoni et al., 2008). The OLD20 is calculated on the basis of the words contained in the English Lexicon Project, a database comprising more than 40,000 words (ELP; http://elexicon.wustl.edu/).

In sum, the OUP influence on word processing remains unclear. However, establishing the significance of OUP in word recognition and reading is important because it can have substantial implications for the manner in which these processes are currently understood.

An essential concern when examining the behavioral effects of a given variable is the potential low sensitivity of the measures commonly used [i.e., response times (RTs) and accuracy]. This problem may be particularly pronounced when word recognition is measured within the lexical decision paradigm because it is difficult to determine the extent to which RTs reflect the time taken to identify a word or to reach the lexical decision itself. The growing popularity of the event-related potential (ERP) technique means that more sensitive measures of cognitive performance are available and used in the study of cognitive performance (Luck, 2005).

Thus, the present study is the first investigation of the neural and behavioral basis of the OUP effect for a set of well-controlled, centrally presented words. Thirteen English native speakers were asked to complete a lexical decision task where forty words were manipulated in terms of their OUP position (i.e., 20 early vs. 20 late) while RTs, response accuracy and ERPs were recorded. If the position of the OUP has an effect on the recognition of words, faster and more accurate processing was expected for those words with an early OUP. This is under the understanding that early OUP words narrow down the lexical search before late OUP words do (Kwantes and Mewhort, 1999). In addition, the neural activity at the N170 will be examined as a component that has been shown to be crucial in visual word identification processes (e.g., Brem et al., 2006; Ellis et al., 2009). It was predicted that if early and late OUP words evoke differing patterns of electrical activity, these differences would be particularly evident on the N170 component.

## MATERIALS AND METHODS

### PARTICIPANTS

Thirteen monolingual, native English-speaking students (five male, eight female) participated in the experiment. All participants were students at Swansea University, had normal or corrected-to-normal vision and were between the ages of 18–25 (mean age: 19) All were rated as strongly right-handed by the Edinburgh Handedness Inventory (Oldfield, 1971). Participants received £15 in return for their participation.

### STIMULI

Experimental stimuli were selected from a modified CELEX database (Baayen et al., 1993). The CELEX database was modified by removing items consisting of more than one word, hyphenated items and words suffixed with –s, –es, and –ed. These were removed so that when OUPs were calculated they would not be affected by plurality, e.g., biscuit would not be compared with biscuits. This left 43,371 words for use as potential stimuli. The OUP for each of these words was calculated by sorting into alphabetical order and, for any given word, comparing the number of contiguous letters-in-position shared with both the preceding word and the following word. The larger of the numbers plus one was the OUP.

From the stimuli pool, a total of forty 7-letter words were chosen. Half of the words had an early OUP (average OUP letter position: 3.65) and the other half had a late OUP (average OUP letter position: 7). Thus, for words, there were two experimental conditions: (1) early OUP words and (2) late OUP words. All words were matched in terms of frequency, bigram frequency, number of syllables, lexical overlap and orthographic neighborhood size and OLD20 values (taken from the ELP). A set of forty 7-letter orthographically legal non-words was also selected from the ARC Non-word Database to act as non-word foils in the lexical decision task (Rastle et al., 2002).

### PROCEDURE

The experiment began with 12 practice trials (six words and six non-words) different from those used as experimental stimuli. Experimental items were presented once the practice trials were over. Participants were exposed to a total of 80 experimental trials (40 words and 40 non-words) upon which they were required to perform lexical decision. Stimuli presentation was randomized and controlled by an IBM Pentium computer, with a 586 processor and 17 inch SVGA display. Participants sat at a viewing distance of approximately 57cm from the display screen in a comfortable chair with a headrest. The experiment was programmed and implemented using E-Prime (2007) software (Psychology Software Tools, 2007). E-Prime (2007) is an experimental generator package that can produce millisecond precision timing.

All stimuli were presented in lower-case, Arial font, size 14 to ensure words were easily readable. Words appeared white against a blue background to minimize screen flicker. Words were presented at fixation and subtended a visual angle of 2°. The central fixation cross subtended a visual angle of 1°.

Each trial commenced with a fixation cross appearing in the center of the screen for 1000 ms. After presentation of the fixation cross, target items were presented for 180 ms at fixation. The participant’s task was to decide, as quickly and as accurately as possible, whether the target stimulus was a real word or not. Participants indicated their responses by pressing a key on a two-key response box. Half of the participants were instructed that the left key indicated a word response and the right key a non-word response. Response keys were reversed for the remaining participants. Once a participant had responded, a message appeared on the screen for 2000 ms indicating that their response had been recorded. Immediately after that, the fixation cross was relit for 1000 ms as the next trial began. The importance of fixating on the cross during the task was emphasized in the pre-experimental instructions, as was the need for speed and accuracy. Participants were also instructed not to blink during trials. During the practice trials, participants were trained in how to time their blinks such that they occurred between experimental trials.

### DATA ACQUISITION

The electroencephalogram (EEG) was recorded in an electrically shielded EEG chamber housed within the Department of Psychology, Swansea University, UK. Participants sat in a comfortable seat, at a viewing distance of 57 cm from the screen, and were instructed to refrain from moving, blinking, or making eye movements during experimental trials. Data were recorded from 64 Ag/AgCl electrodes (BioSemi Active II System, BioSemi Systems, Amsterdam, NL) mounted on an electrode cap and arranged according to the extended International 10–20 system. Sampling rate was 500 Hz and a 0.1–30 Hz bandpass filter was applied. Data were converted off-line to the average reference and analyzed using BESA Research 5.3. (BESA GmbH, 2010). Eye movements were not specUpon completion of the experimental testing session, participants performed an eye movement calibration task for use in eye artifact rejection following the method proposed by Berg and Scherg (1991).

### DATA PRE-PROCESSING

The continuous EEG for each participant was divided into epochs of 1000 ms in length, beginning 200 ms pre-stimulus onset. Trials contaminated with eye artifacts or with peak-to-peak potential differences larger than 75 μv in any channel were rejected. All epochs were baseline-corrected over the 200 ms pre-stimulus interval and converted to the average reference.

**FIGURE 1:**
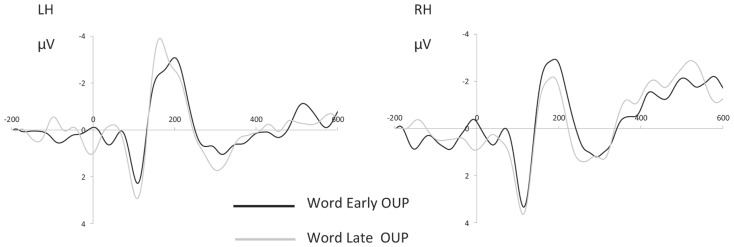
**ERP curves for early and late OUP words recordedover the LH (electrode group consisting of P03, P07, P7) and RH (P04, P08, P8).** Negative is plotted up. Horizontal axis is in milliseconds.

As others (e.g., Schendan and Maher, 2009) standard ERP guidelines were followed to ensure the validity of the analyses (Picton et al., 2000). A criteria of a minimum of 10 artifact free trials per condition was established to ensure that the ERP averages for P1 and N170 were detectable. Grand average ERP curves, plotted for early and late OUP words in each hemisphere electrode group are presented in **Figure 1**.

## ANALYSIS

### BEHAVIORAL RESULTS

Response times of less than 150 ms or more than 2.5 standard deviations from the mean were treated as outliers and removed from the analysis (4.3% of all trials). Eight percent of responses were participant errors and were rejected from subsequent analyses. Non-words were included in the present experiment so as to make lexical decision possible. As it is not possible to manipulate the OUP of non-words, data for non-words will not be analyzed. Mean RTs, standard deviations and accuracy rates for words and non-words are presented in **Table 1**.

**Table 1:** Mean response times (M), standard deviations (SDs) and percentage accuracy [Acc (%)] as a function of orthographic uniqueness point.

	Words		Non-words
	Early OUP	Late OUP	
M	379	350	379
SD	172	154	142
Acc (%)	78	87	92

*Descriptive data for non-words is also presented.*

A main effect of OUP was evident in the RT data. Words with a late OUP were recognized significantly faster than those with an early OUP: *F_1_*(1,12) = 8.94, MSe = 5479.86, *p *< 0.01, $\eta_{p}^{2}$ = 0.43, *F_2_*(1,38) = 4.41, MSe = 13816.81, *p *< 0.05, $\eta_{p}^{2}$ = 0.10.

In the by-subjects analysis of response accuracy, the advantage for late OUP words was observed again. By-subjects, late OUP words were recognized more accurately than early OUP words: *F_1_*(1,12) = 13.45, MSe = 508.65, *p *< 0.005, $\eta_{p}^{2}$ = 0.53. The by-items analysis showed no main effect of OUP on response accuracy.

### ERP RESULTS

**FIGURE 2:**
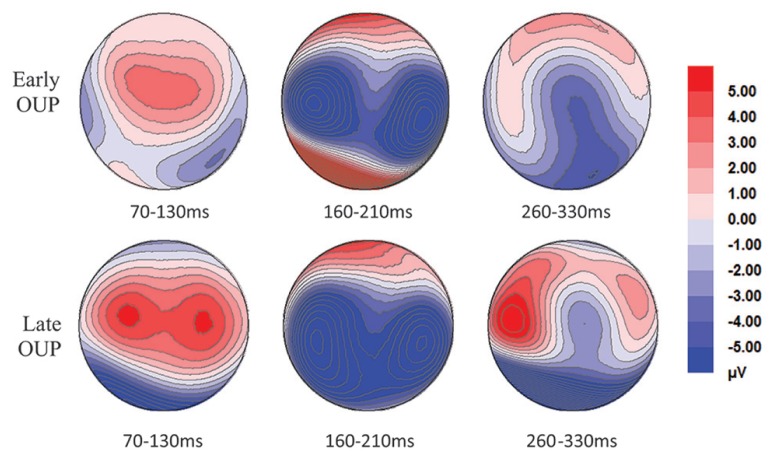
**Topographic scalp maps for early and late OUP words**.

Only trials with correct responses were included in ERP analyses. Grand average RMS curves, plotted for all conditions across all electrodes, indicated three prominent peaks in the ERP distribution, at ~100, ~170, and ~300 ms post-stimulus onset. Due to the fact that the average RT in the behavioral task was 365 ms the peak occurring at ~300 was considered to be too close to decision time. Therefore analyses focused on P1 and N170. These components were defined after examining grand average topographies as the maximal positive deflection between 70 and 130 ms (P1) and the maximal negative deflection between 160 and 210 ms (N170) over parietooccipital sites. Analyses were focused on two groups of electrodes, formed from the average of PO3, PO7, and P7 over the left hemisphere and PO4, PO8, and P8 over the right hemisphere. As others (e.g., Schendan and Maher, 2009) standard ERP guidelines were followed to ensure the validity of the analyses (Picton et al., 2000). A criteria of a minimum of 10 artifact free trials per condition was established to ensure that the ERP averages for P1 and N170 were detectable. Grand average ERP curves, plotted for early and late OUP words in each hemisphere electrode group are presented in **Figure 1**. Topographic scalp maps for early and late OUP words are presented in **Figure 2**. 

#### P1

At 100 ms, amplitudes over the RH were slightly larger than those over the LH, although this effect only approached significance: *F*(1,12) = 3.48, MSe = 4.19, $\eta_{p}^{2}$ = 0.23, *p* = 0.08. There was no main effect of OUP: *F*(1,12) = 3.01, MSe = 339.22, $\eta_{p}^{2}$ = 0.20, n.s., and no interaction of hemisphere and OUP: *F*(1,12) = 2.20, MSe = 446.32, $\eta_{p}^{2}$ = 0.16, n.s.

#### N170

There were no main effects of either OUP or hemisphere at 170 ms on mean amplitudes. However, these factors interacted: *F*(1,12) = 7.84, MSe = 5.01, *p* < 0.05, $\eta_{p}^{2}$ = 0.42. Bonferroni-corrected *post hoc* comparisons were used to determine the nature of the interaction. Early OUP words evoked voltages of equal magnitude in both hemispheres. For late OUP words, amplitudes recorded over the LH (-3.1 μv) were significantly more negative than those recorded over the RH (-1.85 μv; *p* = 0.01). This can be seen in **Figure 1**.

No main effects of OUP or hemispheres were observed in the peak latency analysis. However, OUP and hemisphere interacted again: *F*(1,12) = 10.88, MSe = 961.62, *p* < 0.01, $\eta_{p}^{2}$ = 0.50. In the RH, early and late OUP words achieved peak voltage at similar latencies; in the LH, activity evoked by late OUP (174 ms) words peaked significantly faster than that for early OUP words (191 ms; *p* = 0.02).

## DISCUSSION

The aim of the present study was to determine the effect of OUP on both behavioral and electrophysiological responses. Participants performed lexical decision on centrally presented letter strings with early and late OUPs whilst EEG recordings were made. Standard behavioral measures of RT and accuracy were obtained, in addition to ERP measures of mean amplitude and peak latency.

The behavioral results are clear: words with a late uniqueness point were recognized faster and more accurately than those with an early uniqueness point. Analysis of ERPs demonstrated differences on the N170 component between early and late OUP words both within and across hemispheres. In the LH, at 170 ms, late OUP words achieved peak latency significantly earlier than early OUP words. Across hemispheres, early OUP words generated equivalent activity in both the LH and the RH, whilst late OUP words generated larger negativities over the LH than the RH at 170 ms.

The results from the experiment presented here are consistent with those of Miller et al. (2006) in suggesting that when words are matched in relevant lexical variables – including total lexical overlap and orthographic similarity (OLD20) – there is a consistent processing advantage for late OUP words over early OUP words. The present results are also in line with other findings such as those observed by Lamberts (2005) in relation to OUP and those reported by Lima and Inhoff (1985) in relation to lexical constraint. The majority of these studies (Lima and Inhoff, 1985; Miller et al., 2006) employed a sentence-reading paradigm where parafoveal information played a crucial role. The results of the current research extend understanding in this area by demonstrating that a facilitatory effect for late OUP words is also found in tasks involving the identification of single words.

The difference between the findings reported here, a 29 ms benefit for late OUP words over early OUP words, and those of Kwantes and Mewhort (1999), who observed a 26 ms advantage for early over late OUP are possibly attributable to the way stimuli were matched in terms of lexical variables. Specifically, stimuli in the present research were matched in terms of the extent to which each target shared four letters-in-position in common with other words following Lamberts (2005) suggestions in addition to be controlled for the more recent measure of orthographic similarity (i.e., OLD20). The results of the present experiment show that when word sets share the same lexical characteristics (e.g., orthographic similarity, frequency) an effect of late OUP words is apparent under conditions of central presentation. Kwantes and Mewhort (1999) account of left-to-right sequential processing of centrally presented words predicts faster recognition times for words with an early OUP. The results of the present experiment do not support such an account.

The present study represents the first electrophysiological evidence of an effect of OUP on neural activity. Interestingly, early and late OUP words generated distinctly different patterns in each of the hemispheres on the N170 component. The behavioral advantage for late OUP words was reflected in the ERP findings in two ways: firstly, in the peak latency analysis, where, in the LH, late OUP words achieved peak latency significantly earlier than early OUP words and, secondly, across hemispheres, where late OUP words generated larger responses over the LH than the RH.

Considering that ERP responses to early OUP words were of equal magnitude in both hemispheres, the behavioral facilitation observed for late OUP words may have been driven by LH activity. This may be due to the fact that, for a late OUP target, the OUP falls to the right of fixation, whereas, for an early OUP target, the OUP falls either at, or slightly left of, fixation. It is well-established that words presented entirely to the RVF are identified faster and more accurately than those in the LVF [see Ellis (2004) for a review]. Studies that explore visual field asymmetries typically displace stimuli between 2 and 3° from fixation (Bourne, 2006), where contralateral stimulation of the hemispheres is assured (subject to suitable experimental control). Traditionally when studies have used a central presentations of words (between 1 and 2°), bilateral projection of the foveal region has been assumed (Garey et al., 1991). Recently, the bilateral representation view has been challenged on the basis of behavioral (e.g., Lavidor et al., 2001; Ellis et al., 2005) and computational evidence (Shillcock et al., 2000) that suggests that information falling in the foveal region is not bilaterally represented but instead the central visual field is split through the vertical midline, with contralateral projection occurring for targets displaced immediately to the left and right of fixation. According to the split fovea theory, the crucial information comprised in the words with late OUPs was being systematically projected to the LH, whereas the information from words with early OUP, which fell at or slightly left of fixation, was projected to either the RH of both hemispheres.

Although the present findings do not support a word recognition account that is strictly sequential, they are neither indicative of a pure parallel processing of printed words. The observed faster responses for late OUP words could be understood as a partial parallel processing that operates in an “ends-in” scanning manner. If analysis of the word is based on an “ends-in” scan, this would mean that a word with an OUP at the last letter (i.e., a late OUP word), would be perceived before than a word with the OUP in the middle of the word (i.e., an early OUP word). This is if we consider that the “ends-in” scanning manner processes the end of the word (and the very beginning) before it gets to the middle.

In addition, the interaction observed in the N170 between OUP and hemispheres indicated a differential hemispheric processing of words with early and late OUPs, with late OUP words showing larger amplitudes and peaking earlier in the left hemisphere. These hemispheric differences shown in the processing of centrally presented words could imply that recognizing words is a hybrid product of the parallel mechanisms argued to reside in the left hemisphere with the more serial processing manner claimed to be characteristic of the right hemisphere (Ellis et al., 2009). Similarly, these findings can also be accommodated within the remits of the SERIOL model and result from differences in the way orthography is encoded in each hemisphere with faster timing of firing of those units initially processed by the LH (Whitney, 2001, 2008).

The present study shows that the issue of the manner of processing in word recognition is complex. The observed differential intervention of the two hemispheres and the processing advantage found for late OUP implies that word recognition might not be operated in a pure serial or parallel manner but as a mixture of both processing mechanisms.

## Conflict of Interest Statement

The authors declare that the research was conducted in the absence of any commercial or financial relationships that could be construed as a potential conflict of interest.
